# The social paradoxes of commercial surrogacy in developing countries: India before the new law of 2018

**DOI:** 10.1186/s12905-020-01087-2

**Published:** 2020-10-15

**Authors:** Virginie Rozée, Sayeed Unisa, Elise de La Rochebrochard

**Affiliations:** 1 Institut National d’Etudes Démographiques (INED), 9 cours des Humanités, F-93300 Aubervilliers, Paris, France; 2 International Institute for Population Sciences (IIPS), Mumbai, India; 3 CESP, Univ. Paris-Sud, UVSQ, INSERM, Université Paris-Saclay, Villejuif, France

**Keywords:** Surrogacy, Gender, Survival strategy, Dirty work, India, Developing countries

## Abstract

**Background:**

Commercial surrogacy is a highly controversial issue that leads to heated debates in the feminist literature, especially when surrogacy takes place in developing countries and when it is performed by local women for wealthy international individuals. The objective of this article is to confront common assumptions with the narratives and experiences described by Indian surrogates themselves.

**Methods:**

This qualitative study included 33 surrogates interviewed in India (Mumbai, Chennai and New Delhi) who were at different stages of the surrogacy process. They were recruited through five clinics and agencies. This 2-year field study was conducted before the 2018 surrogacy law.

**Results:**

Surrogates met the criteria fixed by the national guidelines in terms of age and marital and family situation. The commitment to surrogacy had generally been decided with the husband. Its aim was above all to improve the socioeconomic condition of the family. Women described surrogacy as offering better conditions than their previous paid activity. They had clear views on the child and their work. However, they declared that they faced difficulties and social condemnation as surrogacy is associated with extra-marital relationships. They also described a medical process in which they had no autonomy although they did not express complaints. Overall, surrogates did not portray themselves as vulnerable women and victims, but rather as mothers and spouses taking control of their destiny.

**Conclusions:**

The reality of surrogacy in India embraces antagonistic features that we analyze in this paper as “paradoxes”. First, while women have become surrogates in response to gender constraints as mothers and wives, yet in so doing they have gone against gender norms. Secondly, while surrogacy was socially perceived as dirty work undertaken in order to survive, surrogates used surrogacy as a means to upward mobility for themselves and their children. Finally, while surrogacy was organized to counteract accusations of exploitation, surrogates were under constant domination by the medical system and had no decision-making power in the surrogacy process. This echoes their daily life as women. Although the Indian legal framework has changed, surrogacy still challenges gender norms, particularly in other developing countries where the practice is emerging.

## Background

Commercial surrogacy is a highly controversial issue, especially when it takes place in developing countries and when it is performed by local women for wealthy international individuals [1–4]. It may indeed deepen gender, class and race inequalities and contribute to the stratification of reproduction [5], favoring the reproduction of rich people while “*depriving or outlawing the mother-work of others*” p. 469 [6]. In social science literature, particularly in feminist literature, there have been heated controversies on transnational and commercial surrogacy with rhetoric focusing mainly on choice, agency and the commodification of women’s bodies and motherhood [7–11]. For some feminist scholars, particularly the radical and materialist feminists, it is considered as an economic non-choice for poor women and appears as coercion by poverty [12–14]. On the contrary, other feminists approach it as a possible “reproductive choice” in a context of poverty where few other options are available for women to live in better conditions [15–17]. Some feminists also criticize the commodification of women’s reproductive body, turning women into disposable beings, living tools or baby machines, and draw parallels with prostitution and slavery [18, 19]. Others view commercial and transnational surrogacy in the same light as other jobs that strengthen economic and racial exploitation, such as outsourced care activities [15, 20]. From an essentialist feminist perspective, commercial surrogacy, whether local or transnational, denies the natural bond between the mother and the fetus and ignores the maternal love created through pregnancy, therefore degrading women and mothering [21–23]. This special bond and love are considered incompatible with market relations. Conversely, other analyses emphasize that surrogates, like any pregnant woman, can distance themselves physically and emotionally from the pregnancy and the fetus [20, 24, 25]. Thus, two opposite positions emerge: one position that clearly opposes surrogacy and demands national or even international prohibition, and another position that argues that legal regulation of surrogacy as labor would more effectively protect women from exploitation [7].

In this contradictory context, India is a textbook case. This country decided to prohibit transnational and commercial surrogacy in December 2018 through the Surrogacy (Regulation) Bill 2016 [26]. India was still considered as the top destination for commercial and transnational surrogacy, with an estimate of more than 12,000 babies born through transnational surrogacy in India [27]. However, facing scandals first in India and then in Asian countries such as Thailand and Nepal [28], the lower house of the Indian Parliament, the Lok Sabha, decided to convert surrogacy into an altruistic, domestic and relational practice. Henceforward, surrogacy can only be carried out by a close Indian relative of a married Indian couple, without any financial compensation. The public argument put forward claimed that this new law aimed to protect women from international exploitation and to preserve the image of the country from the degrading perspective of women’s exploitation through surrogacy (*The Hindu*, October 23, 2015). However, there is no consensus on the positive impact of this law. Researchers such as Rudrappa and associations such as Sama point out the perverse effect of the official wish to protect women [28, 29], arguing that this change could promote a black market and lead to invisible coercion of women by their family and relatives (see *LiveLawin*, January 5, 2019). In addition, other laws have been voted in India to protect women from violence and exploitation without having a major effect on the endemic rapes, domestic violence, femicides and acid attacks [30].

While India withdraws from commercial and transnational surrogacy, new international destinations are emerging, particularly in developing countries [31, 32]. This trend nourishes the related political, ideological and moral controversies regarding the choice of women considered as vulnerable to commit to surrogacy, the possible moral condemnation, the position of the surrogate concerning the future child they bear and the surrogates’ experiences during the medical process. In order to go beyond these political, ideological and moral positions and to avoid *“speculations”* and *“predictions”* [33], these different controversies need to be confronted with empirical data, particularly with the narratives and experiences of the protagonists themselves. Some authors, such as Zsuzsa Berend, argue that the floor must be given to women who engage in surrogacy, to explore the *“way of making sense of surrogacy”* [34]. Daisy Deomampo stated that:

*“Indian surrogates may be, or may become, victims in the unequal relationships formed between surrogate and doctor or intended parent; nonetheless, [she] contend[s] that reliance on the image of the oppressed surrogate neglects the local voices and perspectives long sought by ethnographers and feminists”* p. 173 [35].

The few empirical data existing on surrogacy in developing countries indeed show that this biomedical practice is a very complex issue, oversimplified by public debates and feminist controversies. This is particularly true in field studies conducted in India [13, 15, 16, 36–39], showing that the binary approach to surrogacy (exploitation vs. emancipation) does not systematically reflect social reality, especially the reality described and lived by the surrogates themselves.

The aim of this study is to analyze the experiences of surrogates living in India in 2013–2014 before the prohibition of commercial and transnational surrogacy. We were interested to find out if existing controversies in the scientific literature regarding commercial surrogacy in developing countries such as India were echoed by reports from surrogates themselves.

## Methods

In 2013–2014, we conducted a 2-year field study in three Indian cities (Mumbai, New Delhi, Chennai). We contacted 37 specialized clinics and agencies to seek authorization to conduct interviews with surrogates working for them. Only five clinics and agencies agreed to put us in touch with their surrogates. Through them, we were able to interview, between June 2013 and September 2014, 33 Indian surrogates: 16 in Mumbai (Maharashtra), 15 in Chennai (Tamil Nadu) and 2 in New Delhi (Delhi). All women who were acting or applying as surrogates were eligible for the study whatever their stage in the surrogacy process. We interviewed all the surrogates introduced to the researcher. The recruitment of clinics and agencies was particularly difficult and we cannot rule out the possibility that those who agreed to participate could be those with the best practices [40]. Although the five clinics and agencies were in three different cities and had different organizations (for recruitment, medical care and follow-up), surrogates’ narratives do not appear to differ from one city or clinic to another. In addition, the narratives collected in this study are consistent with those reported in other studies carried out in India [16, 35, 39, 41].

Face-to-face interviews were conducted by a female European researcher (the first author of this article). She spoke in English whereas the surrogate responded in her own language (Hindi, Marathi or Tamil). Direct translation was performed during the interview by a translator, a female Indian student we recruited and trained for the study, or by a medical professional from the clinic or agency. In most cases, a medical professional attended the interview. This condition was imposed by the clinic or agency director. This is a limitation of the study as it may have affected the spontaneity of the surrogates’ narratives. Nevertheless, the researcher who conducted the interviews felt that surrogates expressed themselves with relative freedom, as is reflected in the statements of some surrogates who expressed criticism of the clinic or agency.

Interviews took place directly in the clinic or agency, or in the place where the surrogate lived during pregnancy (at her own home or temporary apartment). Interviews followed a topic guide including five key themes approached according to the stage of the surrogacy process (see supplementary electronic material): social characteristics of the surrogate and her family; her reasons and motivations for becoming a surrogate; her personal experience of surrogacy so far; her experience and relationship (if any) with intended parents; and the reactions and feelings of her husband, relatives and neighborhood. In a few interviews, some issues could not be approached because of time limitations or interruptions. Some surrogates discontinued the interview because they felt tired or uncomfortable, or because the interviews took place in a festive season (such as Holi or Ganesh Chaturthi) and they were impatient to go and celebrate with the other surrogates and medical professionals. These limitations also account for the varying lengths of the interviews (from 12 to 80 min). Interviews were recorded when the surrogate gave explicit oral consent (*n* = 26/33) and directly annotated (hand-written notes) when the surrogates refused (*n* = 7/33). To avoid bias of oral spontaneous translation during the interview, all recordings were afterwards translated afresh from the original language of the surrogate and transcribed into English by an independent translator.

Based on the English version of the transcription, two researchers (the first and last authors) separately annotated and coded the 33 interviews following the inductive and deductive approach used in qualitative data analysis [42]. They then compared their annotations and coding to reach consensus. All three authors used this material to conjointly analyze social paradoxes of commercial surrogacy in India. Because translations of the original interviews were used, no discourse analysis was performed. The quotations from the interviews cited in the present analysis illustrate the surrogates’ statements but cannot be considered as a word-to-word translation. In order to statistically describe surrogates’ narratives, we also coded some items covered in most interviews as quantitative variables, such as comparison of surrogacy with previous job, acceptance of surrogacy in society, surrogacy commitment as a secret, and intended use of money. These quantitative variables make it possible to contextualize the surrogates’ experiences and to provide a dual approach through considering the singularity of each surrogate’s journey by means of the narratives (qualitative approach) and placing them in the broader context of the entire sample interviewed (quantitative approach).

In parallel with surrogates’ interviews, we also conducted interviews with Indian and international parents (*n* = 8) recruited through the same five clinics and agencies and with medical doctors, lawyers, agency managers, association managers and experts (*n* = 32). However, we focus here on interviews with surrogates and make use of the other interviews only to provide a better understanding or a counterpoint to surrogates’ narratives.

## Results

### Profiles of the surrogates

Details of the surrogates interviewed are presented in Table 1. Their sociodemographic characteristics are summarized in Table 2. Surrogates were between 21 and 33 years old. The majority were married and living with their husband (*n* = 26/33). Seven surrogates were no longer living with their husband: two were divorced, two were widows, two were separated but not officially divorced, and one stated that she was a single mother. The 33 surrogates interviewed had between one and three children. The majority of surrogates (*n* = 28/33) thus met the criteria fixed by Indian government guidelines at that time (married, 21–35 years old, with at least one child and less than five live births).

**Table 1:** Profiles of the 33 surrogates interviewed

Surrogate Number	Pseudo	Age Group (yrs)	Number of children	Marital status^**(a)**^	Education level^**(b)**^	Occupation^**(c)**^	Stage of surrogacy process	Previous surrogacy
S01	Vidya	30–33	2	Married	Secondary	Housewife	During pregnancy (7 months)	No
S02	Nafeesa	30–33	1	Separated	-- ^(d)^	Homeworker	During pregnancy (6 months)	No
S03	Sushmita	21–24	2	Married	Illiterate	Homeworker	During pregnancy (4 months)	Yes
S04	Meera	30–33	2	Married	Primary	Private service	During pregnancy (8 months)	No
S05	Chanda	25–29	3	Married	Secondary	Housemaid	During pregnancy (8 months)	No
S06	Priya	–	–	Married	–	–	During pregnancy (7 months)	–
S07	Jyoti	21–24	1	Married	Primary	Housewife	During pregnancy (5 months)	No
S08	Asma	25–29	1	Divorced	Primary	Homeworker	During pregnancy (3 months)	No
S09	Nichita	–	1	Married	Secondary	Nursing	During pregnancy (7 months)	No
S10	Kajal	25–29	1	Married	Graduate school	Hotel/restaurant worker	During pregnancy (8 months)	No
S11	Cheryl	25–29	1	Widow	Illiterate	Housemaid	After delivery (3 months later)	No
S12	Namrata	25–29	2	Married	–	Housewife	Before pregnancy (awaiting ET ^(e)^)	No
S13	Aditi	21–24	2	Married	–	Housewife	After delivery (10 months later)	No
S14	Kuchi	30–33	2	Married	Primary	Housemaid	Before pregnancy (awaiting ET)	No
S15	Prachi	21–24	1	Married	Secondary	Housewife	Before pregnancy (awaiting ET)	Yes
S16	Sarah	–	2	Married	Primary	Housemaid	Before pregnancy (recruitment)	No
S17	Nidi	25–29	1	Widow	Secondary	Factory worker	During pregnancy (2 months)	No
S18	Rati	30–33	1	Married	Secondary	Factory worker	During pregnancy (3 months)	No
S19	Pushpa	30–33	1	Married	Graduate school	Private service	Before pregnancy (awaiting IVF)	No
S20	Ritika	25–29	2	Married	Secondary	Private service	Before pregnancy (awaiting 2nd ET)	No
S21	Devika	30–33	3	Married	Secondary	Housewife	Before pregnancy (awaiting 2nd ET)	No
S22	Anjali	25–29	3	Married	Primary	Housewife	Before pregnancy (awaiting IVF)	No
S23	Nisha	30–33	3	Married	Primary	Factory worker	Before pregnancy (recruitment)	Yes
S24	Susheela	25–29	2	Married	Graduate school	Hotel/restaurant worker	Before pregnancy (awaiting IVF)	No
S25	Ananda	25–29	2	Divorced	Graduate school	Housemaid/Job-seeker	Before pregnancy (awaiting IVF)	No
S26	Indira	30–33	2	Married	Secondary	Housewife	Before pregnancy (awaiting 2nd ET)	No
S27	Sabina	25–29	2	Single	Secondary	Private service	During pregnancy (1 month)	No
S28	Devna	25–29	2	Married	Secondary	Nursing	During pregnancy (2 months)	No
S29	Simran	21–24	2	Separated	Secondary	Housemaid	Before pregnancy (recruitment)	No
S30	Kasi	21–24	3	Married	Primary	Housewife	Before pregnancy (awaiting IVF)	No
S31	Neela	25–29	2	Married	Primary	Private service	Before pregnancy (awaiting IVF)	Yes
S32	Dipti	25–29	1	Married	Primary	Housewife	After delivery (1 month later)	No
S33	Hasina	30–33	2	Married	Secondary	Housewife	After delivery (3 months later)	No

^(a)^Separated = married but no longer living with the husband

^(b)^Education level = primary (up to 7th); secondary (8th to 11th); high school (from 12th)

^(c)^Private service = security in a shopping mall, receptionist in a construction company, water supply company, jewelry salesperson, newsagent employee

^(d)^- = missing data

^(e)^*ET* Embryo transfer

**Table 2:** Sociodemographic characteristics of surrogates interviewed

	n/N^**(a)**^	%	95% CI^**(b)**^
**Age (years)**			
21–24	6/30	20	[8–39]
25–29	14/30	47	[29–65]
30–33	10/30	33	[18–53]
**City of recruitment**			
Chennai	15/33	45	[29–63]
Mumbai	16/33	48	[31–66]
New Delhi	2/33	6	[1–22]
**Marital status**			
Single (separated, divorced, widow)	7/33	21	[10–39]
Married (living with husband)	26/33	79	[10–39] [61–90]
**Number of children**			
1	11/32	34	[19–53]
2	16/32	50	[32–68]
3	5/32	16	[6–34]
**Education level**			
Illiterate	2/29	7	[1–24]
Primary (up to 6th)	10/29	34	[19–54]
Secondary (7th to 11th)	13/29	45	[27–64]
Graduate school	4/29	14	[5–33]
**Professional activity**			
Housewife	11/32	34	[19–53]
Wage-paid employment	21/32	66	[47–81]
**Monthly family income (in Indian rupees)**			
< 10,000	5/13	38	[15–68]
10,000	3/13	23	[6–54]
> 10,000	5/13	38	[15–68]
**Religion**			
Hindu	22/31	71	[52–85]
Muslim	5/31	16	[6–34]
Christian	4/31	13	[4–31]

^(a)^For each item, the number (N) of surrogates who answered the question is indicated so that the item could be coded

^(b)^As per recommendations for the analysis of small sample data, confidence intervals were estimated using the Wilson score interval with continuity correction [43]. Contrary to confidence intervals based on the classic approximation by Gaussian law, the Wilson score intervals are non-centered confidence intervals

Regarding their education level, the majority (*n* = 17/29) had been to school at least until secondary school. Two surrogates stated that they were illiterate while four stated that they had high school and postgraduate education. The majority of surrogates (*n* = 21/32) had been in wage-paid employment, mainly in the service industries, before committing to surrogacy. The surrogates’ family income varied widely, between 2000 and 30,000 INR [30 to 420 euros^1^] per month, with a median income of 10,000 INR [140 euros].

Most of the women were surrogates for the first time (*n* = 28/32). Regarding the stage of the surrogacy process at the time of interview (Table 3), 14 were pregnant, four were interviewed after delivery and 15 were not yet pregnant (being recruited or receiving medication, awaiting embryo transfer or egg retrieval through in vitro fertilization). Most women were or had been egg donors before becoming surrogates (*n* = 15/18). As in India surrogacy is gestational surrogacy only, the surrogate never provides her egg for the future child she bears (the surrogates’ retrieved eggs are used for a couple other than the one for whom they are bearing the child).

**Table 3:** Surrogates’ experiences and motivation

	n/N^**(a)**^	%	95% CI^**(b)**^
**Stage of surrogacy at the interview**			
Awaiting agreement and pregnancy	15/33	45	[29-63]
During pregnancy	14/33	42	[26-61]
After delivery	4/33	12	[4-29]
**Previous experience as a surrogate**			
Yes	4/32	13	[4-30]
No	28/32	88	[70-96]
**Previous experience as an egg donor**			
Yes	15/18	83	[58-96]
No	3/18	17	[4-42]
**Main reason for surrogacy commitment**			
Financial	32/33	97	[82-100]
Other	1/33	3	[0-18]
**Comparison of surrogacy with previous job**			
Surrogacy is better paid	14/21	67	[43-85]
Surrogacy offers better working conditions	5/21	24	[9-48]
No comparison made	2/21	10	[2-32]
**Would recommend her daughter to become a surrogate**			
Yes	7/13	54	[26-80]
No	6/13	46	[20-74]
**Would recommend surrogacy to another woman**			
Yes	17/18	94	[71-100]
No	1/18	6	[0-29]
**Would repeat the surrogacy experience**			
Yes	3/15	20	[5-49]
No	12/15	80	[51-95]
**Surrogate thinks that surrogacy is an unaccepted practice in the Indian society**			
Yes	30/30	100	[86-100]
No	0/30	0	[0-14]
**Surrogate will keep surrogacy commitment secret**			
Yes	28/33	85	[67-94]
No	5/33	15	[6-33]
**Expressed desire to see the child at birth or to have some news**			
Yes	10/21	48	[26-70]
No	11/21	52	[30-74]
**Expressed difficulties or apprehension about being a surrogate**			
Yes	18/32	56	[38-73]
No	14/32	44	[27-62]
**Why did surrogate think that intended parents would choose / had chosen her (sevezral possible answers)**			
Physical criteria	14/26	54%	[34-73]
Already a mother and a wife	12/26	46%	[27-66]
Personality	5/26	19%	[7-40]
Reproductive history	6/26	23%	[10-44]
Chance or the will of God	4/26	15%	[5-36]
**Intended use of money (several possible answers)**			
Children	17/33	52%	[34-69]
Regular expenses	14/33	42%	[26-61]
Pay debts	10/33	30%	[16-49]
Get the family its own home	9/33	27%	[14-46]
Other	5/33	15%	[6-33]
**Had a preference regarding intended parents (age, marital status, religion or origins)**			
Yes	3/21	14%	[4-37]
No	18/21	86%	[63-96]
**Who suggested surrogacy**			
Friends and/or family	17/33	52%	[34-69]
Media, TV	11/33	33%	[19-52]
Broker	3/33	9%	[2-25]
Other	2/33	6%	[1-22]
**Who took the decision for surrogacy commitment**			
Family or marital decision	26/30	87%	[68-96]
Surrogate alone	4/30	13%	[4-32]

^(a)^For each item, the number (N) of surrogates who answered the question is indicated so that the item could be coded

^(b)^As per recommendations for the analysis of small sample data, confidence intervals were estimated using the Wilson score interval with continuity correction [43]. Contrary to confidence intervals based on the classic approximation by Gaussian law, the Wilson score intervals are non-centered confidence intervals

### Making the decision to become a surrogate

Information on surrogacy practice was first provided to the woman by friends and family (*n* = 17/33) or by the media (*n* = 11/33) in Chennai (Table 3). Surrogacy was suggested by brokers in only three cases (surrogates S14, S16, S23, see Table 1), indicating that brokers have now been replaced by word of mouth, as some medical doctors explained to us.

Surrogates usually considered (*n* = 26/30) that the decision to become a surrogate was taken collectively with their family (husband, mother-in-law). The husband was not systematically consulted. For instance, Nafeesa (S02) explained that her husband had a second wife. Initially, he did not want her to become a surrogate, but she told him that as he had a second wife without consulting her, he didn’t have to decide for her. She nevertheless asked her mother for her approval. Moreover, four surrogates made the decision alone (S11, S25, S27, S29).

Four surrogates (S03, S16, S22, S23) had to convince their husband, who at first refused. Two of these women (S22, S23) later separated from their husbands. Their husbands had concerns about surrogacy because they considered that it could be dangerous for their wife’s health, because of what people would say, or because they were not familiar with the medical in vitro fertilization process and at first associated surrogacy with adultery. Surrogates need the approval of their husband because his signature is required by medical doctors (if there is no husband, the contract can be signed by another relative such as the mother or a sister).

### Facing poverty but above all a desire for social upward mobility

Nearly all surrogates clearly stated that money was their primary motivation for committing to surrogacy (*n* = 32/33)^2^ (Table 3). Surrogates had earned or would earn between 200,000 and 400,000 INR. This represents 30 months of family income, as the median family income of the surrogates was 10,000 INR.

Some of the surrogates (*n* = 10) stated that their financial condition was critical as they had very heavy debts to repay (from 4 to 10 lakhs INR, i.e. 5600 to 14,000 euros) or because their husband was no longer working for health reasons. For these women, surrogacy could be a “non-choice” and two of them stated that only poor women who suffer become surrogates:

*“Money is the first aspect of respect. It [surrogacy] is not accepted because it is associated with poor people. Who else comes to become a surrogate?”* Simran (S29)

However, most surrogates declared that they had no specific financial problems but needed extra money:

“*More money! And if the body can help [someone]!*” Nisha (S23)

“*Who doesn’t want more money in this world?*” Prachi (S15)

Surrogates showed clear intentions as to how the money earned would be used (Table 3). It was intended for the surrogates’ own children (*n* = 17/33), for day-to-day expenses (*n* = 14/33), to pay family debts (*n* = 10/33) or to get the family its own place to live (*n* = 9/33).

Half of the women would not advise their daughter to become a surrogate (*n* = 6/13 surrogates interviewed on this issue). They explained that they became surrogates so that their daughter would not need to do so:

*“Financial problems have to stop with me”* Devika (S21)

If the daughter became a surrogate that would mean, according to them, that she also was in a financial crisis (S21, S27). Answers to the question *“would you recommend surrogacy to your daughter in the future?”* attest to the desire for upward social mobility.

### A clear position concerning the child

Surrogates did not express any specific difficulties in facing the separation from the child they were carrying, nor were they expecting a role in the child’s future.

Surrogates denaturalized their work. They declared that they were only providing a space in their body for the child to grow (Meera, S04), explaining that:

“*My stomach is housing a child*” Asma (S08)

“*It’s like renting a car*” Pushpa (S19)

Some surrogates declared:

“*I only want to do the job*” Namrata (S12)

“*The job is done*” Dipti (S32) and Hasina (S33)

Nevertheless, 10 surrogates declared that they would like or would have preferred to have some news, to see the child or at least to have a photo, just to be sure that the product of their work, the child, was healthy and looked happy (Table 3). This demand was rarely met by medical doctors who usually took the child just after delivery. The doctors explained that this was a way to avoid attachment of the surrogate to the child she delivered and to limit possible blackmail toward intended parents.

### Surrogacy approached as a physical job

Surrogacy was usually described as a positive experience. Most surrogates (*n* = 17/18) would recommend it to another woman (Table 3). However, the majority (*n* = 12/15) declared that they would not repeat the experience because the medical procedures were physically too painful and it was too hard living away from family and children (for those who were away from home during pregnancy). Surrogacy was indeed perceived as a “one-time shot”:

“*A one-off opportunity*” Pushpa (S19)

“*A parenthesis in life*” Meera (S04)

“*An incident that happened in life*” Asma (S08)

Although Ananda (S25) stated that she had “*no fears, only interests*”, nearly half of the surrogates (*n* = 18/32) declared that they had some apprehension or difficulties mainly related to delivery and cesarean delivery. The narratives on job difficulties thus largely related to the physical aspects.

Similarly, according to some surrogates, intended parents chose them on the basis of physical criteria (*n* = 14/26) mainly because they looked healthy, or because of their personality (*n* = 5/26), such as being cooperative, their status as a mother and wife (*n* = 12/26) or their reproductive history (having healthy children, previous delivery without complications) (*n* = 6/26). Simran (S29) had difficulty in finding intended parents because she did not meet physical criteria: she was considered too thin and not able to carry a possible twin pregnancy.

### Surrogacy better than other jobs

Of the 21 surrogates who had previously been in wage-paid employment (Table 2), the majority (*n* = 19/21) emphasized that surrogacy was a preferable option to their previous job (Table 3). They declared that this activity was better paid (*n* = 14/21) and had better working conditions (*n* = 5/21).

Sabina (S27) explained that when she was working in a jewelry shop, she used to go back home late (after working extra non-paid hours) and drivers used to harass her. Another surrogate, Devna (S28), declared that her previous job, in a hospital, was more tiring and stressful. Kajal (S10), who had worked in catering, declared that she was regularly harassed and that there was not this kind of abuse with surrogacy. Nichita (S09) explained that in her previous job in a hospital, she had to take care of and serve others, whereas now other people were taking care of her.

Overall, surrogates explained that:

“*It is not a difficult job, you just need to rest*” Kuchi (S14).

For the first time in their life, surrogates had peaceful days, they did not have to manage a home, they did not have to serve anyone, and they received full attention from medical doctors, professionals, their husband and rich intended parents. It was unusual for them to benefit from such extra care.

### Facing social condemnation of surrogacy

Surrogates are generally kept away from their family and neighborhood. Among the women who were pregnant (*n* = 14), eight were in a surrogacy home, two were staying in their own home, and four were in temporary accommodation (with the intended parents, in a hostel, or in a private room rented by the intended parents).

Surrogates (*n *= 30/30) stated that among their relatives and in their neighborhood, surrogacy was associated with extra-marital relationships (Table 3) which are totally illicit in India. Some surrogates declared that they preferred to stay away from their family and children during pregnancy to preserve the reputation of their family and husband. The majority of surrogates (*n* = 28/33) had told no one (except close family) about their surrogacy commitment (Table 3). They explained to their family that they had to leave the city for employment purposes. Those who were staying at home generally planned to say that they were expecting their own child, and then that something had happened to the baby.

Surrogates were convinced that they were not doing anything wrong but, according to them, society thought differently. They explained that Indian people are quick to pass judgement but:

*“They don’t feed you”*, *“They won’t help you”* Asma (S08)

*“People judge it badly but nobody helps”* Kasi (S30)

The majority of surrogates wanted greater awareness in society. This demand could explain why they welcomed our study, hoping that it would spread more information on surrogacy.

The surrogates explained that people who condemn them for doing sinful, dirty work are only ignorant, and so are not entitled to judge them. Two women explained that educated people understand and accept the process of surrogacy, but illiterate people think it is immoral (S19, S27). Some of them declared that surrogacy allowed them to do something good in their life: helping a childless couple whom they thought were in distress, or being a better mother by investing in their own children’s future.

### Under domination with no autonomy

Surrogacy is sealed by a contract between surrogates, intended parents and medical doctors. In our study, 23 surrogates had signed the surrogacy contract.^3^ Eight surrogates had not read the agreement (because it was in English), but they declared that it had been thoroughly explained to them. The majority of surrogates (*n* = 12/15) did not keep a copy. According to medical doctors, no copy was left with the surrogates for confidential reasons (concealment of the intended parents’ identities). Surrogates explained that they had to do everything the doctors asked them to do. This condition was sometimes written into the agreement.

Surrogates did not choose the intended parents, and they made no demand to do so. The majority of surrogates (*n* = 18/21) declared that they had no preference regarding the age, marital status, religion or nationality of the intended parents:

“*I can serve anybody*” Ananda (S25).

Only one surrogate, Devna (S28) explained that she was wondering how the intended parents would behave toward her as they belonged to a higher caste. Some surrogates declared that they were aware of hierarchical relationships. Some criticism was expressed of the relationship with doctors because surrogates *“are poor and illiterate”* (S03, S29, S30). Others criticized medical doctors’ lack of confidence in them, for instance not allowing them to see the child after birth.

However, no criticism was expressed regarding the organization of surrogacy. Despite difficulties, surrogates described their ongoing or past experience as a positive one. They felt they acquired new knowledge and competencies. Kasi (S30) declared for instance that, through surrogacy, she developed some skills such as communication skills through the numerous appointments with medical doctors and agency managers. This positive experience may also be linked with the medical care, unrelated to pregnancy, that some of them received, like Sushmita (S03) who had eye surgery to correct a squint.

## Discussion

Based on surrogates’ narratives, we present new insights into Indian women’s lived experience of how surrogacy impacts upon their own perceptions of their role as women, spouses and mothers, revealing a complex reality. On the one hand, surrogates’ narratives echo existing criticisms expressed in the literature and in political debates regarding lack of autonomy, economic exploitation and need of money. On the other hand, however, their narratives simultaneously reveal a consciousness of their working conditions and social situation, and some empowerment and benefits of being surrogates. In the remainder of the discussion, we describe how our findings can be seen as pivoting on four paradoxes, echoing the surrogacy controversies in feminist literature. The first paradox is related to the issue of “non-choice” in committing to surrogacy. The second deals with the low moral standards associated with surrogacy in India, giving the women’s feedback and experience of this occupation. Thirdly, another paradox concerns the emotional and bodily involvement of surrogates*.* Finally, we showed that surrogacy as it is experienced and described relates to specific gender norms in India. Our study findings validate some preconceptions and deconstruct others, revealing social and gender paradoxes in surrogacy practice in developing countries such as India.

### The paradoxes of economic non-choice

The first paradox deals with the reasons why women commit to surrogacy. It is commonly assumed that in developing countries where surrogacy is commercial, only poor women in immediate need of money commit to it. In our field study, most surrogates interviewed did not appear to be in desperate poverty. The median income of the surrogates interviewed was 10,000 INR [140 euros] which was well above the Indian family median income (5500 INR per month, or around 80 euros)^4^ [44]. They clearly stated their financial motivation, but they saw the practice as a unique opportunity for upward social mobility and described better working conditions than their previous job. A similar middle-class profile of surrogates has already been described in two other studies [15, 36]. Overall, Indian surrogates appeared to be on an upwardly mobile social trajectory both in our study and in other research [45].

The financial motivation for surrogacy observed in our study is in line with all other studies in India, as well as with studies in Israel where it is also paid [25]. This is in contrast with some other countries. In the United Kingdom, surrogacy is unpaid [46]. In the United States, surrogates are paid but they rarely acknowledge financial considerations as their main motivator [34, 47], and Heather Jacobson even showed that they find such an idea offensive [48].

In our study, the majority of surrogates had previously been in wage-paid employment whereas in the general Indian population, only 42% of women are in paid employment [49]. Surrogates tended to describe surrogacy as a paid activity offering better conditions than their previous job. In our field study as in Rudrappa’s study [15], surrogates explained that working late and reaching home late may be frowned upon in their neighborhood and lead to harassment or a bad reputation. Some authors have observed that other employment may indeed be more harmful, dangerous and abusive, with a much lower income than surrogacy [4, 23, 50, 51].

Facing the need for money and sometimes financial crisis and debts, women had to find strategies, not necessarily to survive but to live better. Surrogacy appears as an option to earn a large sum of money that would be impossible with any other work. They considered that using their reproductive body was a better alternative than other wage labor available for women in India.

Surrogacy thus appears as a strategic and thoughtful choice to improve living conditions. Our empirical data do not sustain the hypothesis that it is an economic non-choice for surrogates. A question that remains unexplored is the impact of surrogacy on the surrogates’ life. Does it lead to better economic and daily life conditions? More social studies are needed, especially over the long term, to evaluate the impacts of such a practice on surrogates’ living conditions, on their children’s future and on their conditions as women.

### The moral paradoxes

The second paradox concerns the moral condemnation of surrogacy. In the “public imagination”, surrogacy is considered as immoral because it is associated with commercialization of motherhood and with sex work [52]. In India, a patrilineal society, married women are traditionally assigned to their husband, family and home and their body is supposed only to ensure lawful descent for the family [53, 54]. The moral condemnation contrasts with surrogates’ own representations of surrogacy. The surrogates we interviewed rejected the stigma, valorizing their commitment and condemning the condemners.

Surrogacy is not an accepted practice in the surrogates’ community and may lead to stigmatization [2, 3, 38, 55]. Surrogacy would reveal the family’s need of money. More generally, for reasons of respectability, women’s wage-paid employment outside the home is not socially well accepted in India. Remunerated activities outside the home are socially perceived as degrading for women. The fact that the majority of the surrogates interviewed were working before commitment to surrogacy attests on the one hand that some of them were in need of money, but on the other hand it also reveals the specific profile of these women as they were already countering the stigma attached to women’s employment.

Stigmatization is not necessarily specific to surrogacy, but it is intensified here as the woman’s body is directly involved. People are not aware of new reproductive technologies and would therefore think that surrogates had to have a sexual relationship with a man other than their husband. Amrita Pande drew a parallel between surrogacy and “dirty work” [13], based on Everett Hughes’ research [56]. “Dirty work” is defined as tasks that are physically, socially or morally tainted [57]. Moral taint occurs when an occupation is regarded as somewhat sinful or of dubious virtue, which is the case of surrogacy.

When surrogates explained that people were not familiar with the process of in vitro fertilization, they placed themselves apart. They now knew that it was possible to have babies without sexual intercourse and they were aware of medical advances, which was not the case of their relatives. They felt that by becoming surrogates they were becoming part of a modern and knowledgeable society, exactly as was observed with institutionalized delivery and use of modern contraception [58].

The surrogates explained that people who condemn them for doing sinful, dirty work are only ignorant, and so are not entitled to judge them. “*Condemnation of the condemners*” is a classic mechanism observed among dirty workers to counterbalance the social stigma of their job [57]. Surrogates developed strategies to combat the stigma attached to surrogacy, to overcome its negative representation and to valorize their activity.

### The paradox of the “labor of love”

A third paradox appears regarding the maternal bond. As noted in the introduction, some scholars, especially essentialist feminists, point out that there is an inevitable attachment of pregnant women to the fetus. The surrogates described a clear affective but detached attitude to the future child, and defined surrogacy as a physical activity more than an emotional one.

We observed no kind of maternal bond, which is consistent with other findings in India [44] and observations in other countries such as Canada, the US and the United Kingdom [24]. The bond with the future child that surrogates bear was nevertheless described as an affective bond. It appears more as a nanny bond [25]. However, as in Lamba’s study [44], surrogates declared that they were taking special care for this pregnancy, more than when they were awaiting their own child, because the future baby represented an important investment for the intended parents and a large sum of money for them.

Elizabeth Anderson compared surrogacy to a “*labor of love*” and explained that surrogacy is not very different from *“many already accepted practices which separate genetic, gestational, and social parenting, such as artificial insemination by donor, adoption, wet-nursing, and day care”* [22]. For Eileen Boris and Rhacel Parreñas, surrogacy is an intimate labor [59]. They drew a parallel between nannies and surrogates, explaining that both are women whose work involves psychological and bodily intimacy.

The surrogates we interviewed approached surrogacy as a physical job. They disembodied their belly, as in Teman’s study in Israel [25]. Teman showed that Israeli surrogates separated their body from their self and bonded better with the intended mother than with the child they were bearing, through a relationship described as fusional.

### The paradoxes of gender constraints

Surrogacy is often described in the literature as reinforcing gender inequalities. Indeed, we observed that women become surrogates in order to respond to gender constraints as mothers and wives. However, paradoxically, by becoming surrogates they go against gender norms through which women are assigned to one man (the husband), one family (family-in-law), and are not encouraged to have wage-paid employment. Indian women are assigned to maternity, children and the household [13, 54].

Surrogacy was perceived by the women we met as a new way to generate income, without creating suspicions or threatening the reputation of the family (as the surrogacy was kept secret) and thus to take care of their family. At the same time, surrogacy appears as a respite from women’s workload. Surrogacy stems simultaneously from gender constraints and from the will to go beyond gender constraints.

Through our interviews with surrogates, intended parents and medical doctors, we confirm the lack of autonomy, liberty and decision-making power of surrogates regarding pregnancy, delivery and the entire process, as has been observed in other studies conducted in India [60] but unlike findings in other countries (the UK, Israel, the USA). In their narratives, surrogates did not complain of lack of autonomy. We cannot rule out that this absence of complaints was biased by the conditions of interview, since a person from the clinic or agency was often present and since the clinics may have selected surrogates who were considered as having had a trouble-free experience and no specific complaints. However, similar observations were made in other studies such as that of Rudrappa, who interviewed surrogates in surrogacy homes where the presence of cameras in the dormitory was not perceived by the women as disturbing or violating intimacy [15].

The lack of complaints is consistent with gender norms and with women’s daily life in India: Indian women are permanently under the control of their family and husband, with limited decision-making power and limited mobility [61, 62]. Surrogates are constantly under supervision, exactly as they are in their everyday life. Thus, lack of autonomy does not seem to be specifically related to surrogacy, but to Indian society in general.

This domination and submissiveness are reinforced by the fact that surrogates belong to a lower class than medical doctors and intended parents, in a society that is traditionally strongly hierarchical and unequal, where power and autonomy depend on social class and economic resources [63]. The constant control and supervision also echo the strong biomedical power in India over both women and women’s bodies, as already argued by other authors [35, 38, 60]. This medical power and domination are not specific to surrogacy: in India, as elsewhere, medical power is generally exercised over economically disadvantaged and socially marginalized people [64], including in maternity and gynecology services [65].

Medical doctors and intended parents tend to counteract the image of exploitation. For them, surrogacy is a win-win situation: childless couples go back home with a child while surrogates earn a large sum of money. Medical doctors explained that they kept the surrogates away from their homes to protect them from stigmatization, while preparing the women for a better life by organizing English or computer lessons, as is clearly shown in Gudenus’s documentary in Anand [66]. Intended parents stated that they had chosen the clinic that offered the best ethical and medical conditions in order to avoid possible exploitation [67].

In this context, however, and as far as it can be judged based on our interviews, surrogates were not forced to enter into the surrogacy process. We had the impression that they did not want to portray themselves as submissive women, nor as vulnerable women and victims. They described themselves as women taking control of their destiny, taking the few options available to fulfil their responsibilities as spouses and mothers. These women have deconstructed the usual image of Indian surrogates as vulnerable and exploited women. Surrogacy clearly shows *“how women both exert power and are subject to it”* [35].

## Conclusions

In feminist literature, surrogacy is described either as a survival strategy, as dirty work denigrated by the women’s peers, as exploitation by medical doctors and intended parents, or as a reproductive right and an opportunity for upward social mobility. Empirical data make it possible to go beyond the theoretical field. From our research based on surrogates’ narratives, the reality of surrogacy in India appears to lie between the two extremes and to embrace these antagonistic features.

As in other developing countries where commercial surrogacy is flourishing, India has a significant reserve of reproductive workers because of gender norms and gender inequality that generate unfavorable conditions for women [41]. Surrogacy may be a way to improve the living conditions of the family and children. At the same time, transnational and commercial surrogacy threatens patriarchal and traditional family systems: women’s wombs are no longer assigned to their family-in-law and to male descent. Thus, behind the narrative of women’s rights protection, the prohibition of commercial and transnational surrogacy [26] can be analyzed as a way to preserve the traditional family and gender norms.

The economic, social and political context, especially the gender norms, of a given society may lead to possible paradoxical situations, including for women themselves. It is commonly assumed that women in developing countries are vulnerable, and that they are forced to commit to sex work or outsourced reproductive labor because of economic and gender constraints [17, 20, 68]. However, women cannot be reduced to a “*status of pure alienated victim*” p. 53 [69]: this would deny their resilience and their capacity to act and to decide, even in a context of power and domination.

The social and especially the gender paradoxes that we have analyzed here with transnational and commercial surrogacy echo the social paradoxes of globalization related to migration, sex work and care activities [70, 71]. Globalization has created new social and economic opportunities for women, but at the same time it has strengthened global and local gender inequalities. For example, Robin Cavagnoud has explored families of Bolivian women who had migrated alone for economic reasons and worked in care activities. He showed that families valorize the new role of these women as breadwinners but they also consider them as “bad mothers” for having abandoned their own children. Likewise, the family remains organized around a female and maternal figure (such as the grandmother) and not around the father who did not migrate [72].

Care activities, such as childcare, and domestic work or sex work are generally performed by economically disadvantaged local women or immigrant women. The choice to become a nanny, a domestic employee or a sex worker may rely on “flawed consent” p. 71 [73]. But such commitment gives them more economic resources as well as greater autonomy and decision-making power as women, mothers and wives. However, in doing so, they face class and sex domination, moral resistance and stigmatization [68, 74, 75].

Gender paradoxes are therefore not specific to surrogacy, but rather are common to women’s outsourced activities in global society. Interestingly, they seem to be exacerbated when these activities directly deal with the body of poor women, as is the case of surrogacy.

## Supplementary information

**Additional file 1.** Guidelines for interviews with surrogates.

## Data Availability

Audio recordings of the surrogates interviewed and their transcription in English are not publicly available to protect the privacy and anonymity of the Indian surrogates. Table 1 presents characteristics of the 33 surrogates interviewed, each one being identified by a pseudo (fake first name given by the authors). The raw data are available upon reasonable request from the corresponding authors after having obtained the relevant legal and ethical authorizations.

## Footnotes

1 Exchange rate 2009–2014 (constant over this period, 1 INR corresponding to 0.014 euros)

2 The one surrogate (Neela, S31) who declared that money was not the first motivation explained that she wanted to be a surrogate because she lost the baby at the first surrogacy attempt and also because she had realized the suffering of infertility (a friend of hers had committed suicide because of infertility).

3 Intended parents were mainly Indians in the Chennai agency and mainly internationals in the other clinics or agencies of the study. For transnational contracts, the surrogates we interviewed were aware that the intended parents were foreigners but they did not systematically know their nationality.

4 The national urban median income is 9100 INR (130 euros) and the national rural median income is 4300 INR (60 euros).

## References

[1] Crozier GK (2014). Too blunt a tool: a case for subsuming analyses of exploitation in transnational gestational surrogacy under a justice or human rights framework. Am J Bioeth.

[2] Deonandan R, Loncar M, Rahman P, Omar S (2012). Measuring reproductive tourism through an analysis of Indian ART clinic websites. Int J Gen Med.

[3] Kirby J (2014). Transnational gestational surrogacy: does it have to be exploitative?. Am J Bioeth.

[4] Orfali K, Chiappori PA (2014). Transnational gestational surrogacy: exploitative or empowering?. Am J Bioeth.

[5] Colen S, Ginsburg FD, Rapp R (1995). “Like a mother to them”: stratified reproduction and west Indian child care workers and employers in New York. Conceiving the New World order: the global politics of reproduction.

[6] Rapp R (2001). Gender, body, biomedicine: how some feminist concerns dragged reproduction to the Center of Social Theory. Med Anthropol Q.

[7] Borrillo D (2019). Disposer de son corps : un droit encore à conquérir. Paris: éditions Textuel.

[8] Gupta JA (2006). Toward transnational feminism: some reflections and concerns in relation to the globalization of reproductive technologies. Eur J Womens Stud.

[9] Majumdar A (2014). The rhetoric of choice: the feminist debates on reproductive choice in the commercial surrogacy arrangement in India. Gend Technol Dev.

[10] Neyer G, Bernardi L (2011). Feminist perspectives on motherhood and reproduction. Hist Soz Forsch.

[11] Parks JA, Scott Sills E (2016). Gestational surrogacy and the feminist perspective. Handbook of gestational surrogacy international clinical practice and policy issues.

[12] Donchin A (2010). Reproductive tourism and the quest for global gender justice. Bioethics.

[13] Pande A (2014). Wombs in labor: transnational commercial surrogacy in India.

[14] Saravanan S (2016). ‘Humanitarian’ thresholds of the fundamental feminist ideologies: evidence from surrogacy arrangements in India. J Gend Fem Stud.

[15] Rudrappa S (2015). Discounted life: the Price of global surrogacy in India.

[16] Sama Resource Group for Women and Health (2012). Birthing a market: a study on commercial surrogacy.

[17] Sera JM (1997). Surrogacy and prostitution: a comparative analysis. Am Univ J Gend Soc Pol Law.

[18] Corea G (1985). The mother machine: reproductive technologies from artificial insemination to artificial wombs.

[19] Spar DL (2006). The baby business: how money, science, and politics drive the commerce of conception.

[20] Satz D (1992). Markets in Women's reproductive labor. Philos Public Aff.

[21] Agacinski S (2009). Corps en miettes.

[22] Anderson E (1990). Is women’s labor a commodity?. Philos Public Aff.

[23] Rothman BK (2014). The legacy of patriarchy as context for surrogacy: or why are we quibbling over this?. Am J Bioeth.

[24] Busby K, Vun D (2010). Revisiting the Handmaid's tale: feminist theory meets empirical research on surrogate mothers. Can J Fam Law.

[25] Teman E (2010). Birthing a mother: the surrogate body and the pregnant self.

[26] Indian Parliament (2016). The surrogacy (regulation) bill n°257 of 2016.

[27] Shetty P (2012). India’s unregulated surrogacy industry. Lancet.

[28] Nadimpally S, Banerjee S, Venkatachalam D (2016). Commercial surrogacy: a contested terrain in the realm of rights and justice.

[29] Rudrappa S (2017). Reproducing dystopia: the politics of transnational surrogacy in India, 2002-2015. Crit Sociol.

[30] Marius K (2017). L’inde : la loi avance, le patriarcat résiste. Trav Genre Soc.

[31] Scott Sills E (2016). Handbook of gestational surrogacy. International clinical practice and policy issues.

[32] Torres G, Shapiro A, Mackey TK (2019). A review of surrogate motherhood regulation in south American countries: pointing to a need for an international legal framework. BMC Pregnancy Childbirth.

[33] Pande A (2010). Commercial surrogacy in India: manufacturing a perfect mother-worker. Signs (Chic).

[34] Berend Z (2016). The online world of surrogacy.

[35] Deomampo D (2013). Transnational surrogacy in India: interrogating power and women's agency. Frontiers.

[36] Deomampo D (2016). Transnational reproduction. Race, kinship, and commercial surrogacy in India.

[37] Majumdar A (2017). Transnational commercial surrogacy and the (un) making of kin in India: Oxford University press.

[38] Saravanan S (2013). An ethnomethodological approach to examine exploitation in the context of capacity, trust and experience of commercial surrogacy in India. Philos Ethics Humanit Med.

[39] Vora K (2013). Potential, risk, and Retrun in transnational Indian gestational surrogacy. Curr Anthropol.

[40] Rozée V (2017). La gestation pour autrui en Inde : des difficultés de terrain révélatrices d’une réalité controversée. J Anthropol.

[41] Rudrappa S (2012). Working India’s reproduction assembly line: surrogacy and reproductive rights. West Humanit Rev.

[42] Paillé P, Mucchielli A (2016). L'analyse qualitative en sciences humaines et sociales.

[43] Wilson EB (1927). Probable inference, the law of succession, and statistical inference. J Am Stat Assoc.

[44] Lamba N, Jadva V, Kadam K, Golombok S (2018). The psychological well-being and prenatal bonding of gestational surrogates. Hum Reprod.

[45] Rozée V, Unisa S, Rochebrochard L (2019). Sociodemographic characteristics of 96 Indian surrogates: are they disadvantaged compared with the general population?. PLoS One.

[46] Jadva V, Imrie S, Golombok S (2015). Surrogate mothers 10 years on: a longitudinal study of psychological well-being and relationships with the parents and child. Hum Reprod.

[47] Ragoné H (1994). Surrogate motherhood: conception in the heart.

[48] Jacobson H (2016). Labor of love. Gestational surrogacy and the work of making babies.

[49] International Institute for Population Sciences (2007). Macro international: National Family Health Survey (NFHS-3), 2005–06: India: volume I.

[50] Humbyrd C (2009). Fair trade international surrogacy. Dev World Bioeth.

[51] Ramskold LAH, Posner MP (2013). Commercial surrogacy: how provisions of monetary remuneration and powers of international law can prevent exploitation of gestational surrogates. J Med Ethics.

[52] Pande A (2009). Not an ‘angel’, not a ‘whore’: surrogates as ‘dirty’ Workers in India. Indian J Gend Stud.

[53] Guilmoto C, Kulkarni PM, Gautier A (2004). Les femmes, la caste et l’Etat. Cinquante ans de planification familiale en Inde. Les politiques de planification familiale : cinq expériences nationales.

[54] Marius K (2016). Les inégalités de genre en Inde. Regard au prisme des études postcoloniales.

[55] Bagcchi S (2014). Mothers who turn to surrogacy to support their families face ostracism, study shows. BMJ.

[56] Hughes E, Rohrer JH, Sherif M (1951). Work and the self. Social psychology at the crossroads.

[57] Ashforth BE, Kreiner GE (1999). “How can you do it?” dirty work and the challenge of constructing a positive identity. Acad Manag Rev.

[58] van Hollen C (2003). Birth on the threshold: childbirth and modernity in South India.

[59] Boris E (2010). Parreñas RS (eds.): intimate labors: cultures, technologies, and the politics of care.

[60] Tanderup M, Reddy S, Patel T, Nielsen BB (2015). Informed consent in medical decision-making in commercial gestational surrogacy: a mixed methods study in New Delhi, India. Acta Obstet Gynecol Scand.

[61] Kishor S, Gupta K (2009). Gender equality and Women’s empowerment in India. National Family Health Survey (NFHS-3), India, 2005–06.

[62] Phadke S, Khan S, Ranade S (2011). Why loiter? Women and risk on Mumbai street.

[63] Jaffrelot C (1996). L’Inde contemporaine de 1950 à nos jours.

[64] Cohen P (2008). Figures contemporaines de la santé en Inde.

[65] Qadeer I, Reddy S (2013). Medical tourism in India: perceptions of physicians in tertiary care hospitals. Philos Ethics Humanit Med.

[66] Gudenus V (2013). Ma na Sapna. A Mother’s dream (video documentary).

[67] Rozée V, Unisa S (2015). Surrogacy as a growing practice and a controversial reality in India: exploring new issues for further researches. J Womens Health Issues Care.

[68] Ehrenreich B, Hochschild AR (2002). Global woman: nannies, maids, and sex workers in the new economy.

[69] Falquet J (2008). De gré ou de force. Les femmes dans la mondialisation, vol. le genre du monde.

[70] Falquet J, Hirata H, Lautier B (2006). Les nouveaux paradoxes de la mondialisation. Cah Genre.

[71] Hirata H, Le Doare H (1998). Les paradoxes de la mondialisation. Cahier du Gedisst.

[72] Cavagnoud R, Zavala de Cosio M, Rozée V (2014). El impacto de las migraciones internacionales de mujeres bolivianas en el trayecto de Vida de sus hijos no migrantes: el Caso de El alto. El género en movimiento Familias y migraciones.

[73] Mathieu L (2015). Sociologie de la prostitution.

[74] Agrawal A (2006). Migrant women and work.

[75] Mazumdar I (2006). Women workers and globalisation: emergent contradictions in India.

