# Supplementary material for: The social paradoxes of commercial surrogacy in developing countries: India before the new law of 2018
Source: BMC Womens Health. 2020 Oct 15;20:234. doi: 10.1186/s12905-020-01087-2 (PMC7559454; doi:10.1186/s12905-020-01087-2)
Supplement: Supplementary file 1 — Additional file 1. Guidelines for interviews with surrogates. [file 12905_2020_1087_MOESM1_ESM.docx]

**Guidelines for interviews with surrogates**

Consent form *(presented in English, Marathi or Hindi)*

“A French National Institute (National Institute for Demographic Studies, INED), in collaboration with an Indian Institute (International Institute of Population Sciences, IIPS, Mumbai, India), is currently carrying out scientific research on surrogacy in India. There has been very little scientific research on surrogacy, especially in India. Our aim is to understand surrogacy better by considering the characteristics and experience of women involved in surrogacy. This is why we are asking you to participate in this interview and to share your experiences with us. You are free to accept or not and your decision will have no effect on the medical care you receive nor on the surrogacy process. Interviews are completely anonymous and confidential. You can decide to stop or discontinue the interview at any time you want. The time that you give to this study is a precious contribution to research to understand the use of surrogacy in India.”

*Before starting, ask the surrogate whether she agrees to the interview being recorded. Reassure her that she can accept or refuse, ask her if she feels comfortable. Tell her that the simultaneous interpreter is here to make the interview easier. Reassure her that the recording will only be listened to and used by the researcher and the assistant, and only for scientific purposes, and that neither the clinic staff nor parents nor any other people will have access to the recorded interview.*

*Semi-structured interviews: The aim is to ask as few questions as possible, to ask questions arising from what the surrogate has already explained or related, while attempting to approach as many of the issues below as possible.*

# Five main issues

## **(1) Social characteristics of the surrogate and her family**

Please tell us about yourself, about your history…

### Origins

Where do you come from? Have you always lived there?

Where does your family come from?

### Place of living and family type

Where do you live? What is the name of the place? Is it a slum area?

Have you always lived there?

Who do you live with?

### Education/profession

Did you go to school? When/Why did you stop going to school?

What is/was your occupation?

### Religion

Do you have any religion? Is religion important for you?

### Children

Do you have any children?

Are they boys? girls?

How old are they?

How many children would you like to have? (or would you have liked to have had?) Do you plan to have another child/more children? If not, do you plan to use contraceptive methods?

### Husband (if any)

Please tell us about your husband… His work? His origins?

## (2) Becoming a surrogate: reasons and motivations

Please tell us how you came to be a surrogate…

### Information

How did you get information about surrogacy? about the clinic?

### Decisions

Who took the decision for you to become a surrogate? And why?

What are the reasons that led you to become a surrogate? Are there any reasons?

### Money

Do you know how much you will earn? Have you received all or part of the money?

How do you plan to use the money?

### Comparison

What were the reasons why you chose surrogacy rather than another activity or source of income? Can you compare both activities/sources of income?

## (3) Personal experience of the surrogacy process

### Recruitment

How were you recruited by the clinic?

Can you describe recruitment? Was there an interview with the doctor? Medical tests? How long does it take to become a surrogate?

### Agreement

Did you sign an agreement?

Did you read it?

It was an agreement between who and who? Who was there when it was signed?

What did the agreement say? The main points, do you remember?

Do you have a copy of this agreement?

### Current stage of surrogacy/previous surrogacy

At which stage of the surrogacy process are you? How long have you been pregnant?

Have you been pregnant for another person before/been a surrogate before?

### Experience as a surrogate

How can you describe your experience as a surrogate so far?

Please give us 3-5 positive points and 3-5 negative points about being a surrogate.

Have you always thought/felt this way? Have your feelings changed? Can you please describe a typical day as a surrogate?

Where did you stay/where are you staying during the pregnancy? What happens to your family/children/husband?

Did you stay/are you staying with other surrogates? (if not, did/do you regularly meet the other surrogates?). What did/do you talk about with the other surrogates? Are they different from you (origins, motivations, experience)?

### Comparison with possible other pregnancies

What kind of difference is there between this pregnancy (surrogate pregnancy) and the pregnancies of your own children? Why is it similar/different?

Where did you have your child? Were your pregnancies followed in the same way as your surrogacy pregnancy is followed here?

### Representations

For you, what does “to be a surrogate” mean?

Apart from money, what are the benefits?

Do you think you are doing something good? something that is gratifying and satisfying?

## (4) Experience/Relationship with (current) intended parents.

Now, about the parents…

(*Use the same terms as those used by the surrogate: parents, intended parents, clients…*)

### Characteristics and history

What can you tell us about the parents? What do you know about them? (their marital situation, their nationality, their physical characteristics). Did you choose them?

Are they like you expected or wanted them to be? What would you have preferred?

Why are they using surrogacy?

Why do you think they are using surrogacy in Mumbai? In India (if they are foreigners)?

### Relationship

Have you ever met the parents? When? How many times? How (Internet, face to face)?

Did you want to know/meet them? Would you like to know/meet them? Why?

How would you describe the relationship (if any) with the parents?

Do you plan to be in touch with them in the future? Do you want to? Why?

### Being special

Why do you think they chose you?

## (5) Reaction and feelings of partner, relatives, neighborhood

Now, about your family, community, neighborhood…

### Social representation

How is surrogacy seen in society? Why do you think it is seen like that? Do you mind what people think? What can be done to change their opinions?

What does religion say about surrogacy?

### Disclosure

Have you told your family/neighborhood about surrogacy or not? Why?

Depending on the answer (the surrogate has told/will tell/will not tell):

How did/do/would they react? What was/will be/would be their reaction? What did/do/would they do to you or your family? What happened/will happen/would happen to you or your family? What would happen if people knew what you are doing?

## End of interview: Conclusion

Would you do it again (be a surrogate)?

Would you advise other women to be surrogates? What kind of women? Have you already suggested it to anyone?

If you could change something in your experience as a surrogate, what would you change?

What would you think if your daughter wanted to be a surrogate in the future?

The interview is finished now. Is there anything you’d like to add? A last comment you’d like to make?

**Thank you so much for your help in this study!**
